# Female Sex Hormones Pattern and Its Relation to Disease Severity and Treatment in Pre- and Postmenopausal Patients with Chronic Hepatitis C Virus (Genotype 4) Infection

**DOI:** 10.1155/2015/927974

**Published:** 2015-08-17

**Authors:** Nora H. Ahmed, Taghrid B. El-Abaseri, Hesham F. El-Sayed, Taher I. El-Serafi

**Affiliations:** ^1^Department of Medical Biochemistry, Faculty of Medicine, Suez Canal University, Ismailia 41111, Egypt; ^2^Department of Pediatrics and Unit of Clinical Epidemiology, Faculty of Medicine, Suez Canal University, Ismailia 41511, Egypt

## Abstract

Chronic hepatitis C (CHC) course revealed differences between men and women. Male gender and postmenopausal women are thought to be of the critical factors affecting HCV infection progression. The study aimed to assess female sex hormones and their relation to disease severity and treatment in HCV infected females. Subjects were divided to 2 groups: 44 CHC female patients and 44 controls. Both groups were classified to premenopausal and postmenopausal females. Serum estradiol (E2), progesterone (PRG), and total testosterone (TT) were assessed using chemiluminescent immunoassay. Our results showed that menopausal patients had significantly higher levels of estradiol, total testosterone, and progesterone compared to controls (*P* < 0.001). Reproductive aged patients had lower level of total testosterone compared to menopausal patients (*P* < 0.001). HCV infected females of reproductive age had higher level of progesterone compared to menopausal HCV infected females (*P* = 0.0014). Indicators of disease severity and treatment response were significantly worse in menopausal women compared to reproductive aged women (fibrosis: *P* < 0.001, activity: *P* = 0.045, and treatment: *P* < 0.001). We observed that lower estradiol level may be related to fibrosis severity in CHC females. Higher total testosterone and progesterone levels may be related to fibrosis severity and poor response to treatment in CHC menopausal females only.

## 1. Introduction

Hepatitis C is caused by HCV infection. It is estimated that about 3% of the world's population has been infected with HCV and 170 million are chronic carriers at risk of developing liver cirrhosis and/or liver cancer [1]. Prevalence of HCV in Egypt is 14.7% [2].

The clinical course of chronic hepatitis C revealed several differences between men and women. Male gender is thought to be one of the critical factors in the progression of HCV infection [3]. Besides, the development of hepatic fibrosis is faster in postmenopausal than in premenopausal women [4]. In addition, serum total testosterone is associated with increased risk of both advanced hepatic fibrosis and advanced hepatic inflammatory activity in HCV infected men [5].

Sex differences are believed to be one of the factors affecting response to therapy. Female sex correlated positively with sustained virological response (SVR) [6]. However, early menopause associated with lack of SVR among women with genotype 1 hepatitis C virus infection [7].

## 2. Materials and Methods

### 2.1. Type of Study and Selection of Patients

Descriptive analytical study included 44 female patients, proven to have chronic hepatitis C infection [8] by polymerase chain reaction (PCR) and 44 control females, age matched to the patients, were selected from healthy blood donors, proven to be negative for HCV on hepatitis C antibody test.

Patients were recruited from Virology Center in Ismailia Fever Hospital, a referral center for treatment of HCV in Ismailia under the supervision of the Ministry of Health as part of the national project for combating viral hepatitis. Patients were subjected to thorough history taking, clinical examination, and routine workup including complete blood picture (CBC), serum transaminases (AST, ALT), total bilirubin, albumin, International Normalized Ratio (INR), alpha fetoprotein (AFP), and HCV quantitative PCR. Histopathological examination of histological activity and degree of hepatic fibrosis, of ultrasound guided percutaneous liver biopsy, was performed according to Metavir's score. Blood samples were included in the study between May 2013 and October 2013.

### 2.2. Serum Estradiol, Progesterone, and Total Testosterone Assay

Serum estradiol, progesterone, and total testosterone were assessed using IMMULITE 1000 (Siemens, IMMULITE, catalog number LKE21 for estradiol, LKTW1 for total testosterone, and LKPW1 for progesterone), a solid-phase enzyme-labeled, chemiluminescent immunoassay. Chemiluminescence generates electromagnetic radiation as light. The light intensity depends on a chemical reaction using a labeled enzyme and a chemiluminescent compound supplied as the enzyme substrate in excess to assure saturation kinetics. Light intensity is measured using stored master curves based on manufacturer's instructions.

### 2.3. Patients' Classification

Patients were classified according to their reproductive status to 22 premenopausal and 22 postmenopausal females in each group (patients and controls). Further grouping of female patients according to (1) treatment (combined interferon and ribavirin therapy) response (nonresponders: patients who had detectable HCV RNA at weeks 12, 24, and 48; relapsers: patients who had detectable HCV RNA 24 weeks after stopping treatment; and responders: patients with sustained virological response (SVR) with undetectable HCV RNA six months after stopping treatment), (2) viral load (<600,000 and ≥600,000), and (3) fibrosis stage (≤F2 and >F2) was done to compare between groups.

### 2.4. Patients Characteristics

Females included were nonsmokers, of reproductive age group (i.e., with regular menses, not pregnant or lactating), ≥18 years, and postmenopausal females (i.e., no menstrual period for 12 consecutive months).

CHC females having hepatocellular carcinoma (HCC), coinfection with human immunodeficiency virus (HIV), coinfection with hepatitis B virus (HBV), other causes of liver disease (alcoholic, autoimmune, and Wilson disease), previous liver transplant, hormone replacement therapy, hormonal contraception, and history of active substance or alcohol consumption were excluded.

### 2.5. Patients Consent

All regulations adopted by the ethics committee in Faculty of Medicine, Suez Canal University, including patient consent were considered and approved.

### 2.6. Statistical Analysis

Quantitative parametric variables were presented by mean and standard deviation (SD) and compared by Student's *t*-test. Qualitative variables were compared by the chi-square test. In all tests, *P* value was considered significant if <0.05.

## 3. Results

The demographic, clinical, biochemical, and histopathological data of the studied subjects are shown in Table 1. Similar distributions were noted for age, BMI, age at menarche, marital status, reproductive status and the presence of diabetes and/or hypertension in HCV female patients, at reproductive age and menopausal group, and their respective healthy controls. Among the baseline laboratory data platelets count and albumin levels were significantly lower (*P* = 0.007 and 0.03, resp.), while INR level was significantly higher (*P* = 0.005) in the menopausal patients compared to the reproductive aged patients. Menopausal women had worse indicators of disease severity (fibrosis: *P* < 0.001; activity: *P* = 0.045) and reduced response to treatment (*P* < 0.001) compared to reproductive aged women.

Hormonal levels in the studied subjects showed that estradiol level is higher in menopausal patients compared to their healthy controls (*P* < 0.001) (Table 1). Similarly, menopausal HCV infected females have significantly higher level of total testosterone than controls (*P* < 0.001). Total testosterone level is lower in reproductive aged HCV infected females than menopausal HCV infected females (*P* < 0.001). Progesterone level in HCV infected females of reproductive age group was higher than in menopausal HCV infected females (*P* = 0.0014).

The relation between fibrosis and hormonal levels in reproductive aged and menopausal patients is shown in Table 2. Estradiol level appeared to be higher in the group with fibrosis stage ≤F2 than the group of fibrosis stage >F2 (*P* = 0.005) for reproductive aged patients. In menopausal patients total testosterone and progesterone levels were significantly lower (TT: *P* = 0.002; PRG: *P* = 0.023), while estradiol level was significantly higher (*P* = 0.003) in group with fibrosis stage ≤F2 than the group of fibrosis stage >F2.

The relation between virological response and hormonal levels in the studied groups is shown in Table 3. While the majority of the reproductive aged women responded to HCV therapy, only 13.6% appeared to be nonresponders and 9.1% were relapsers. In menopausal patients, total testosterone and progesterone levels were higher in the nonresponder group than in the responder group (TT: *P* < 0.001; PRG: *P* < 0.001). No *t*-test was done for reproductive aged patients as there were great differences in numbers of each group, which cannot be compared statistically. The study of menopausal patients included one patient who showed relapse in response to treatment with hormonal levels (E2 = 97.3, TT = 29.00, and PRG = 0.60).

Table 4 showed that the comparative relationship of hormonal levels and viral load in both groups (reproductive aged and menopausal females) appeared with no statistically significant value in all variables.  Table 5 showed the correlations between hormonal levels in reproductive aged and menopausal patients and the fibrosis stage, activity grade, and virological response.

Table 6 showed the relation between estradiol level and fibrosis in reproductive aged and menopausal patients; lower estradiol level was associated with significant increase of fibrosis level in reproductive aged patients between 35 and 50 years and menopausal patients between 47 and 55 years (RA: *P* = 0.037; M: *P* < 0.001). The relation between estradiol level and virological response in reproductive aged and menopausal patients (Table 7) showed that estradiol level was significantly lower with nonresponders in menopausal patients between 47 and 55 years (*P* = 0.03).

## 4. Discussion

The findings of our study reveal that CHC infection is associated with elevated levels of estradiol, total testosterone, and progesterone in both pre- and postmenopausal females when compared to their healthy controls. This may be explained by the impaired metabolism of these hormones as a result of the defective liver function in chronic liver disease patients [9–13].

Menopausal HCV infected women had higher levels of estradiol, total testosterone, and progesterone when compared to their healthy controls (*P* < 0.001, <0.001, and <0.001, resp.). On the other hand reproductive aged women had statistically nonsignificant increased levels of these hormones when compared to their healthy controls (*P* < 0.28, <0.346, and <0.80, resp.). Menopausal Greek females with HCC females had also higher levels regarding estradiol and testosterone compared to their healthy controls [13]. To our knowledge this is the first study to evaluate progesterone level in women with HCV infection.

There were higher significant level of total testosterone and lower significant level of progesterone in menopausal women with CHC compared to females of reproductive age with CHC, and this was not the case for estradiol level that showed a slight, however, nonsignificant decrease (*P* = 0.093). On the other hand the comparison between the same groups (menopausal and reproductive aged females) in control women showed higher significant levels regarding estradiol and progesterone levels in reproductive age females (*P* < 0.001, <0.001, resp.) and nonsignificant difference in testosterone level between both groups.

HCV infection is associated with increased hormonal levels of estradiol, total testosterone, and progesterone in both groups reproductive aged and menopausal women; moreover estradiol and progesterone levels decreased with age, while total testosterone level increased with age in HCV group. In addition, although hormonal levels in reproductive aged group were higher than their healthy controls they are of statistically nonsignificant values and appeared to be minimally affected by hepatitis C, while menopausal group had hormonal levels high enough to be statistically significant in all variables compared to their healthy controls, providing evidence that this group was highly affected by hepatitis C.

Our study revealed that women of reproductive age had lower disease severity as measured by the liver necroinflammation activity and fibrosis compared to older menopausal women. The observed changes in the studied hormonal levels could be implicated in these findings. The high estradiol and progesterone levels and low total testosterone level in the reproductive aged patients may in part have protected against the development of severe liver injury. In contrast, menopausal women exhibited greater disease severity, probably due to the decline in estradiol and progesterone levels and the rise in total testosterone level that occurs with menopause development, supporting the concept that the progression of fibrosis in women is not on the same pace: mild during reproductive age and severe after menopause. Similar to our findings, Villa et al. 2012 conducted a study on four groups of women divided according to their reproductive stage (fully reproductive, premenopausal, early menopausal, and late menopausal) in addition to four age-matched groups of men serving as controls [14]. According to this study they found that liver fibrosis was advanced in the early menopausal HCV infected women compared to fully reproductive or premenopausal women. In addition, late menopausal women had higher liver fibrosis compared with younger women [14]. The favorable role of estrogens in liver disease has also been suggested by Shimizu et al., 2001, and Maffei et al., 2004, two case reports, in which different pathological conditions required prolonged estrogen treatment in two young men, one with hepatitis C and the other with nonalcoholic steatohepatitis. In the first case, the addition of estradiol reduced disease activity and maintained viral loads at lower levels than those observed before treatment. In the second case, it reversed hepatic steatosis and insulin resistance [15, 16].

A number of molecular mechanisms could explain the protective role of E2; for example, E2 inhibits the transforming growth factor- (TGF-) b1 expression and hepatic stellate cell (HSC) activation, thereby suppressing the induction of hepatic fibrosis [15–17]. In addition, E2 also downregulates tumor necrosis factor (TNF) alpha, interleukin-6 (IL-6), and IL-1b, mediators contributing to hepatic necroinflammation and the activation of HSCs [18–20]. Moreover, E2 possesses antioxidant, antiapoptotic activities in fibrotic liver and cultured hepatocytes [21, 22] that prevented the accumulation of monocytes-macrophages and inhibits proinflammatory cytokine production, whereas progesterone may counteract the favorable E2 effects [23, 24].

The total testosterone level was significantly higher in menopausal HCV infected females compared to reproductive aged HCV infected females. This finding is in agreement with the recently reported positive correlation between testosterone level and increased risk of both advanced hepatic fibrosis and advanced hepatic inflammatory activity in HCV infected men [5], in which higher unopposed level of testosterone related to higher severity of the disease and increased risk of HCC in men [25]. Other studies did not correlate high testosterone levels to hepatic fibrosis [26]. Potential explanations for the discordant findings of our study and those studies may include differences in underlying target populations, host factors, or difference in viral genotype.

Our findings of excess testosterone-associated risk in HCV positive females are consistent with the findings of increased liver cancer risk in males who abuse anabolic steroids, in androgen-treated Fanconi anemia patients [27], in women with hyperandrogenemia secondary to polycystic ovarian disease [28], and in HBV positive Taiwanese males with higher serum testosterone level [29–31].

To our knowledge no studies addressing the changes in progesterone level have been conducted yet. Our study showed that menopausal patients had poorer responses to combined therapy than reproductive aged patients (menopause versus reproductive age: *P* < 0.001). Previous studies including genotype 1 (most common genotype in Japan and Italy) highlighted the same findings [7, 32, 33].

The relationship between hormonal parameters in menopausal women and virological response showed that higher total testosterone and progesterone levels were significantly associated with unfavorable outcomes (*P* < 0.001 and <0.001, resp.). Estradiol level appeared to be of no significant relationship. A number of previous studies suggested, however, a potential link to estrogen secretion [32, 33]. Another nonconcordant study, the study of Furusyo et al. 2012, used raloxifene hydrochloride (RLX) (oral selective estrogen receptor modulator) plus SOC (standard of care) treatment in postmenopausal women with genotype 1b chronic hepatitis C; the SVR rate was significantly higher for RLX plus SOC patients (61.3%) than for SOC only patients (34.4%) (*P* = 0.0051) [34]. These inconsistent results may be explained by the difference in host genetic factors, viral genotype (Egypt common genotype 4 versus Japan common genotype 1), or ethnicity. Impairments in humoral, cellular, and innate immunity in the elderly and modulation of inflammatory factors, such as IL-6 and TNF-*α*, at menopause may be another important factor which are responsible for decreasing successful responses to treatment in menopausal patients [7, 35].

Our study revealed that there was a statistically nonsignificant difference regarding viral load values between the two groups. Besides, the comparative relationship of hormonal levels and viral load appeared to be of statistically nonsignificant value in all variables of both groups (menopausal and reproductive aged females). This finding is supported by one in vitro study that treated human hepatoma-derived cell line (Huh-7.5 cells) with E2 and PRG and found inhibition of HCV virion production (in case of E2 only) but not HCV RNA replication or HCV protein synthesis [36]. On the other hand, to our knowledge, no studies were found addressing its relation to total testosterone level.

## 5. Conclusion

The present study explored the possible involvement of female sex hormones in the pathogenesis and/or progression of liver disease in HCV infected Egyptian females. We observed that lower estradiol level is significantly related to fibrosis severity in CHC females, while higher total testosterone and progesterone levels are significantly related to fibrosis severity in CHC menopausal females only. Besides, higher total testosterone and progesterone levels are related to poorer treatment response in CHC menopausal females, while estradiol level is not. Additionally, the detected higher hormonal levels did not have significant relation to viral load in both groups. We suggest that screening of progesterone and total testosterone levels should selectively target females with high grade fibrosis. Our findings underscore the potential favorable effects of antitestosterone treatment to target HCV infected females. We assume that giving hormone replacement therapy to infected menopausal females would not have a beneficial effect regarding HCV course or treatment response.

## Supplementary Material

Data sheet for age in menopausal women and estradiol levels in both groups.

## Figures and Tables

**Table 1:** Demographic, clinical, biochemical, and histopathological data of patients and healthy controls.

Variables	Controls (RA)	Cases (RA)	Controls (M)	Cases (M)
*N*	22	22	22	22
Age (yrs)	34.32 ± 8.39	38.27** ± **7.38	57.09 ± 3.73^B^	55.32 ± 3.80^C^
BMI (kg/m^2^)	24.45 ± 3.17	25.14 ± 3.63	27.45 ± 3.23^B^	26.27 ± 3.31
AAM (yrs)	11.41 ± 1.50	12.18 ± 1.62	11.41 ± 1.84	11.18 ± 1.74
Married (%)	14 (63.6%)	18 (81.1%)	21 (95.4%)^B^	21 (95.4%)
Parity	2.29 ± 1.27	2.78 ± 1.11	3.33 ± 1.39^B^	3.38 ± 1.20
DM (%)	3 (13.6%)	3 (13.6%)	10 (45.5%)^B^	7 (31.8%)
HTN (%)	3 (13.6%)	5 (22.7%)	9 (40.9%)^B^	10 (45.5%)
Hemoglobin (12–16 g/dL)	12.43 ± 0.96	12.6 ± 1.2	12.37 ± 1.05	12.52 ± 1.47
TLC (4.000–11.000/mm^3^)	7543.63 ± 2432.9	7081.82 ± 2194.56	7140.45 ± 2241.4	6215.0 ± 2279.34
Platelets (150.000–400.000/mm^3^)	233000 ± 66960.5	214954.5 ± 86489.46	226136.4 ± 59978.8	154818.2 ± 46953.5^A,C^
ALT (40) IU/L	23.68 ± 5.07	51.99 ± 31.93^A^	24.4 ± 3.87	57.86 ± 29.3^A^
AST (40) IU/L	23.18 ± 4.97	48.6 ± 19.87^A^	25 ± 3.48	61.90 ± 27.8^A^
Bilirubin (0–2 mg/dL)	0.63 ± 0.32	0.563 ± 0.22	0.66 ± 0.28	0.66 ± 0.25
Albumin (3.5–5 g/dL)	3.96 ± 0.24	3.9 ± 0.49	3.87 ± 0.28	3.56 ± 0.58^A,C^
INR (0.9–1.1)	0.95 ± 0.2	1.09 ± 0.12	0.91 ± 0.27	1.26 ± 0.24^A,C^
AFP (10 ng/dL)	—	3.56 ± 3.04	—	5.56 ± 3.7
HCV RNA (IU/mL) [baseline before treatment]	—	577340 ± 293440	—	587400 ± 230269
Histological parameters				
≤F2 number (%)	—	16 (72.7%)	—	9 (40.9%)^C^
>F2 number (%)	—	6 (27.3%)	—	13 (59.1%)
<A2 number (%)	—	11 (50%)	—	8 (36.4%)^C^
≥A2 number (%)	—	11 (50%)	—	14 (63.6%)
Response to treatment				
Responders number (%)	—	17 (77.3%)	—	9 (40.9%)^C^
Nonresponders number (%)	—	3 (13.6%)	—	12 (54.4%)
Relapsers number (%)	—	2 (9.1%)	—	1 (4.5%)
Hormonal levels				
E2 (pg/mL)	106.5 ± 50.85	120.0 ± 56.74	20.43 ± 2.12^B^	94.91 ± 46.82^A^
TT (ng/dL)	19.9 ± 0.85	20.51 ± 2.88	19.97 ± 1.02	31.6 ± 13.0^A,C^
PRG (ng/mL)	1.15 ± 0.76	1.21 ± 0.76	0.30 ± 0.133^B^	0.60 ± 0.32^A,C^

A, activity; AAM, age at menarche; ALT, alanine aminotransferase; AFP, alpha fetoprotein; AST, aspartate aminotransferase; BMI, body mass index; DM, diabetes mellitus; E2, estradiol; F, fibrosis; HTN, hypertension; INR, International Normalized Ratio; M, menopausal group; *N*, number of individuals; PRG, progesterone; RA, reproductive age group; TLC, total leucocytic count; TT, total testosterone. *t*-test: ^A^statistically significant difference between corresponding *controls and cases* groups “menopausal or reproductive aged women”; ^B^statistically significant difference between control group reproductive aged women and menopausal women; ^C^statistically significant difference between *HCV infected group* reproductive aged women and menopausal women.

**Table 2:** The relation between fibrosis and hormonal levels in reproductive aged and menopausal patients.

Hormones	Fibrosis stage		Activity grade		Fibrosis stage		Activity grade	
	≤F2	>F2	<A2	≥A2	≤F2	>F2	<A2	≥A2
	(RA)				(M)			
*N* (%)	16 (72.7%)	6 (27.3%)	11 (50%)	11 (50%)	9 (40.9%)	13 (59.1%)	8 (36.4%)	14 (63.6%)
E2 (pg/mL)	139.4 ± 50.72	68.25 ± 37.25^*∗*^	142.67 ± 49.31	97.3 ± 56.54	127.6 ± 44.3	72.3 ± 34.17^*∗*^	113.96 ± 52.29	84.04 ± 41.47
TT (ng/dL)	19.95 ± 1.55	22.0 ± 4.89	21.6 ± 3.67	19.4 ± 1.18	22.1 ± 3.67	38.23 ± 13.09^*∗*^	21.13 ± 3.18	37.64 ± 12.68^*∗*^
PRG (ng/mL)	1.30 ± 0.82	0.97 ± 0.54	1.5 ± 0.8	0.90 ± 0.51	0.407 ± 0.18	0.717 ± 0.34^*∗*^	0.33 ± 0.14	0.74 ± 0.30^*∗*^

^**∗**^Statistically significant difference (*P* < 0.05).

**Table 3:** The relation between virological response and hormonal levels in reproductive aged and menopausal patients.

Hormones	Responders	Nonresponders	Relapsers	Responders	Nonresponders	Relapsers
	(RA)			(M)		
*N* (%)	17 (77.3%)	3 (13.6%)	2 (9.1%)	9 (40.9%)	12 (54.4%)	—
E2 (pg/mL)	120.01 ± 58.49	148.33 ± 38.83	77.35 ± 61.73	106.4 ± 54.4	86.13 ± 42.9	—
TT (ng/dL)	19.6 ± 1.14	22.14 ± 1.67	25.85 ± 8.27	20.21 ± 0.64	40.42 ± 11.43^*∗*^	—
PRG (ng/mL)	1.14 ± 0.77	1.46 ± 0.82	1.41 ± 0.90	0.30 ± 0.11	0.81 ± 0.27^*∗*^	—

^**∗**^Statistically significant difference (*P* < 0.05).

**Table 4:** The relation between viral load and hormonal levels in reproductive aged and menopausal patients.

Hormones	HCV RNA (IU/mL) [baseline before treatment]			
	(RA)		(M)	
	<600,000	≥600,000	<600,000	≥600,000
*N* (%)	10 (45.5%)	12 (54.5%)	10 (36.4%)	12 (63.6%)
E2 (pg/mL)	135.38 ± 58.13	107.18 ± 54.65	106.71 ± 52.69	85.09 ± 41.02
TT (ng/dL)	20.25 ± 1.66	20.73 ± 3.67	29.11 ± 9.99	33.73 ± 15.18
PRG (ng/mL)	1.02 ± 0.64	1.37 ± 0.83	0.55 ± 0.24	0.62 ± 0.38

**Table 5:** The correlations between hormonal levels in reproductive aged and menopausal patients and the fibrosis stage, activity grade, and virological response.

Hormones	Fibrosis stage	Activity grade	Treatment	Fibrosis stage	Activity grade	Treatment
	(RA)			(M)		
E2 (pg/mL)	−0.68	−0.43	−0.29	−0.60	−0.21	0.18
TT (ng/dL)	0.37	−0.35	0.20	0.69	0.74	−0.66
PRG (ng/mL)	−0.04	−0.23	−0.04	0.46	0.62	−0.63

**Table 6:** The relation between fibrosis and estradiol level in reproductive aged and menopausal patients.

	(RA)		(M)	
Age	20–35 yrs *N* (= 9)	35–50 yrs *N* (= 13)	47–55 yrs *N* (= 13)	55–60 yrs *N* (= 9)
	E2 (pg/mL)			
Fibrosis stage				
≤F2	152.1 ± 43.57 *N* (= 7)	129.6 ± 56.11 *N* (= 9)	147.22 ± 23.13 *N* (= 5)	103.15 ± 55.6 *N* (= 4)
>F2	83.0 ± 36.77 *N* (= 2)	60.88 ± 40.56^*∗*^ *N* (= 4)	54.15 ± 13.48^*∗*^ *N* (= 8)	101.26 ± 38.46 *N* (= 5)

Activity grade				
<A2	148.28 ± 51.51 *N* (= 5)	138.00 ± 51.8 *N* (= 6)	129.18 ± 48.78 *N* (= 6)	68.30 ± 41.01 *N* (= 2)
≥A2	122.25 ± 51.78 *N* (= 4)	83.1 ± 57.8 *N* (= 7)	56.31 ± 12.97^*∗*^ *N* (= 7)	111.8 ± 41.01 *N* (= 7)

^**∗**^Statistically significant difference (*P* < 0.05).

**Table 7:** The relation between virological response and estradiol level in reproductive aged and menopausal patients.

	(RA)		(M)	
Age	20–35 yrs *N* (= 9)	35–50 yrs *N* (= 13)	47–55 yrs *N* (= 13)	55–60 yrs *N* (= 9)
	E2 (pg/mL)			
Responders	137.91 ± 55.68 *N* (= 7)	107.48 ± 59.92 *N* (= 10)	116.7 ± 55.4 *N* (= 7)	70.15 ± 43.63 *N* (= 2)
Nonresponders	132.5 ± 38.89 *N* (= 2)	180.00 ± 00.00 *N* (= 1)	58.7 ± 12.4^*∗*^ *N* (= 6)	113.55 ± 45.7 *N* (= 6)
Relapsers	— *N* (= 0)	77.35 ± 61.73 *N* (= 2)	— *N* (= 0)	97.3 ± 00.0 *N* (= 1)

^**∗**^Statistically significant difference (*P* < 0.05).

## References

[1] National Institutes of Health Consensus Development Conference Statement (2002). Management of hepatitis C. *Gastroenterology*.

[2] El-Zanaty F. W. A. (2009). *Egypt Demographic and Health Survey 2008*.

[3] Poynard T., Ratziu V., Charlotte F., Goodman Z., McHutchison J., Albrecht J. (2001). Rates and risk factors of liver fibrosis progression in patients with chronic hepatitis C. *Journal of Hepatology*.

[4] Di Martino V., Lebray P., Myers R. P. (2004). Progression of liver fibrosis in women infected with hepatitis C: long-term benefit of estrogen exposure. *Hepatology*.

[5] White D. L., Tavakoli-Tabasi S., Kuzniarek J., Pascua R., Ramsey D. J., El-Serag H. B. (2012). Higher serum testosterone is associated with increased risk of advanced hepatitis C-related liver disease in males. *Hepatology*.

[6] Ling M. H., Albrecht J., Poynard T., McHutchison J., Goodman Z. (2000). Is an ‘a la carte’ combination interferon alfa-2b plus ribavirin regimen possible for the first line treatment in patients with chronic hepatitis C. The algovirc project group. *Hepatology*.

[7] Villa E., Karampatou A., Cammà C. (2011). Early menopause is associated with lack of response to antiviral therapy in women with chronic hepatitis C. *Gastroenterology*.

[8] (1999). EASL International Consensus Conference on hepatitis C. Paris, 26-27 February 1999. Consensus statement. *Journal of Hepatology*.

[9] Zumoff B., Fishman J., Gallagher T. F., Hellman L. (1968). Estradiol metabolism in cirrhosis. *The Journal of Clinical Investigation*.

[10] Glass S. J., Edmondson H. A., Soll S. N. (1940). Sex hormone changes associated with liver disease. *Endocrinology*.

[11] Bannister P., Oakes J., Sheridan P., Losowsky M. S. (1987). Sex hormone changes in chronic liver disease: a matched study of alcoholic versus non-alcoholic liver disease. *Quarterly Journal of Medicine*.

[12] Maruyama Y., Adachi Y., Aoki N., Suzuki Y., Shinohara H., Yamamoto T. (1991). Mechanism of feminization in male patients with non-alcoholic liver cirrhosis: role of sex hormone-binding globulin. *Gastroenterologia Japonica*.

[13] Mucci L. A., Kuper H. E., Tamimi R., Lagiou P., Spanos E., Trichopoulos D. (2001). Age at menarche and age at menopause in relation to hepatocellular carcinoma in women. *British Journal of Obstetrics and Gynaecology*.

[14] Villa E., Vukotic R., Cammà C. (2012). Reproductive status is associated with the severity of fibrosis in women with hepatitis C. *PLoS ONE*.

[15] Shimizu I., Omoya T., Kondo Y. (2001). Estrogen therapy in a male patient with chronic hepatitis c and irradiation-induced testicular dysfunction. *Internal Medicine*.

[16] Maffei L., Murata Y., Rochira V. (2004). Dysmetabolic syndrome in a man with a novel mutation of the aromatase gene: effects of testosterone, alendronate, and estradiol treatment. *Journal of Clinical Endocrinology and Metabolism*.

[17] Shimizu I., Mizobuchi Y., Yasuda M. (1999). Inhibitory effect of oestradiol on activation of rat hepatic stellate cells in vivo and in vitro. *Gut*.

[18] Rogers A., Eastell R. (2001). The effect of 17*β*-estradiol on production of cytokines in cultures of peripheral blood. *Bone*.

[19] Friedman S. L. (2008). Hepatic stellate cells: protean, multifunctional, and enigmatic cells of the liver. *Physiological Reviews*.

[20] Kireev R. A., Tresguerres A. C. F., Garcia C. (2010). Hormonal regulation of pro-inflammatory and lipid peroxidation processes in liver of old ovariectomized female rats. *Biogerontology*.

[21] Liu Y., Shimizu I., Omoya T., Ito S., Gu X.-S. (2002). Protective effect of estradiol on hepatocytic oxidative damage. *World Journal of Gastroenterology*.

[22] Lu G., Shimizu I., Cui X. (2004). Antioxidant and antiapoptotic activities of idoxifene and estradiol in hepatic fibrosis in rats. *Life Sciences*.

[23] Yuan Y., Shimizu I., Shen M. (2008). Effects of estradiol and progesterone on the proinflammatory cytokine production by mononuclear cells from patients withchronic hepatitis C. *World Journal of Gastroenterology*.

[24] Huang H., He J., Yuan Y. (2008). Opposing effects of estradiol and progesterone on the oxidative stress-induced production of chemokine and proinflammatory cytokines in murine peritoneal macrophages. *Journal of Medical Investigation*.

[25] Tanaka K., Sakai H., Hashizume M. (2000). Serum testosterone: estradiol ratio and the development of hepatocellular carcinoma among male cirrhotic patients. *Cancer Research*.

[26] Nguyen H. V., Mollison L. C., Taylor T. W., Chubb S. A. P., Yeap B. B. (2006). Chronic hepatitis C infection and sex hormone levels: effect of disease severity and recombinant interferon-*α* therapy. *Internal Medicine Journal*.

[27] Velazquez I., Alter B. P. (2004). Androgens and liver tumors: Fanconi's anemia and non-Fanconi's conditions. *American Journal of Hematology*.

[28] Vassilatou E., Lafoyianni S., Vryonidou A. (2010). Increased androgen bioavailability is associated with non-alcoholic fatty liver disease in women with polycystic ovary syndrome. *Human Reproduction*.

[29] Yu M.-W., Chen C.-J. (1993). Elevated serum testosterone levels and risk of hepatocellular carcinoma. *Cancer Research*.

[30] Yu M.-W., Cheng S.-W., Lin M.-W. (2000). Androgen-receptor gene CAG repeats, plasma testosterone levels, and risk of hepatitis B-related hepatocellular carcinoma. *Journal of the National Cancer Institute*.

[31] Yu M.-W., Yang Y.-C., Yang S.-Y. (2001). Hormonal markers and hepatitis B virus-related hepatocellular carcinoma risk: a nested case-control study among men. *Journal of the National Cancer Institute*.

[32] Hoyoshi J., Kishihara Y., Ueno K. (1998). Age-related response to interferon alfa treatment in women vs men with chronic hepatitis C virus infection. *Archives of Internal Medicine*.

[33] Sezaki H., Suzuki F., Kawamura Y. (2009). Poor response to pegylated interferon and ribavirin in older women infected with hepatitis C virus of genotype 1b in high viral loads. *Digestive Diseases and Sciences*.

[34] Furusyo N., Ogawa E., Sudoh M. (2012). Raloxifene hydrochloride is an adjuvant antiviral treatment of postmenopausal women with chronic hepatitis C: a randomized trial. *Journal of Hepatology*.

[35] Ginaldi L., Loreto M. F., Corsi M. P., Modesti M., De Martinis M. (2001). Immunosenescence and infectious diseases. *Microbes and Infection*.

[36] Hayashida K., Shoji I., Deng L., Jiang D.-P., Ide Y.-H., Hotta H. (2010). 17*β*-estradiol inhibits the production of infectious particles of hepatitis C virus. *Microbiology and Immunology*.

